# Epigallocatechin Gallate Promotes Cuproptosis via the MTF1/ATP7B Axis in Hepatocellular Carcinoma

**DOI:** 10.3390/cells14060391

**Published:** 2025-03-07

**Authors:** Yuhan Fu, Lirui Hou, Kai Han, Chong Zhao, Hongbo Hu, Shutao Yin

**Affiliations:** Department of Nutrition and Food Safety, College of Food Science and Nutritional Engineering, China Agricultural University, No. 17 Qinghua East Road, Haidian District, Beijing 100083, China; fuyuhan@cau.edu.cn (Y.F.); houlirui97@163.com (L.H.); hankai1122@cau.edu.cn (K.H.); zhaoch0206@cau.edu.cn (C.Z.)

**Keywords:** EGCG, cuproptosis, copper, ATP7B, MTF1, HCC

## Abstract

Background: Cuproptosis is a form of copper-dependent non-apoptotic cell death. Cancer cells that prefer to use aerobic glycolysis for energy generation are commonly insensitive to cuproptosis, which hinders its application for cancer treatment. Epigallocatechin gallate (EGCG) possesses diverse pharmacological activities. However, the association between EGCG and cuproptosis has not been studied. Methods: The cell viability, proliferation, and cuproptosis-related protein levels were detected to investigate whether EGCG enhances the sensitivity of HCC cells to cuproptosis. The intracellular copper level, related copper metabolism proteins, and gene expression were detected to explore the mechanisms. In addition, a nude mouse xenograft model was established to determine the effects of EGCG on cuproptosis in tumor tissues. Results: The combination of EGCG and copper ionophores significantly enhanced the mortality of HCC cells and heightened the sensitivity of HCC cells to cuproptosis. There was a notable reduction in the expression of copper export protein copper-transporting P-type ATPase (ATP7B). EGCG effectively suppressed metal regulatory transcription factor (MTF1) expression and subsequently hindered the transcriptional regulation of ATP7B. EGCG also facilitated the intratumoral accumulation of copper and augmented susceptibility to cuproptosis in vivo. Conclusions: EGCG can increase the sensitivity of hepatocellular carcinoma cells to cuproptosis by promoting intracellular copper accumulation through the MTF1/ATP7B axis.

## 1. Introduction

Hepatocellular carcinoma (HCC), a primary malignancy of the liver, is one of the most common and lethal cancers worldwide. In Global Cancer Statistics 2020, liver cancer was ranked sixth in incidence and third in mortality in cancer disease globally [1]. The occurrence and development of HCC is a multi-step phenomenon driven by the dysregulated control of gene expression and aberrant activation of basic cellular processes [2]. Molecular targeted therapy and immunotherapy have been demonstrated to have beneficial impacts on advanced-stage HCC [3]. However, patients are susceptible to drug resistance and are insensitive to most drugs. Therefore, it is necessary to develop effective options for HCC to improve its treatment efficacy.

Copper is an essential micronutrient and is required as a cofactor for enzymes to mediate a host of essential physiological and biochemical reactions, including mitochondrial respiration, redox balance, and the biosynthesis of melanin, collagen, and neurotransmitters [4]. Excessively elevated copper levels lead to cytotoxicity. In 2022, a new form of non-apoptotic cell death was proposed and defined as “cuproptosis”, which is triggered by copper binding to lipoylated protein, like dihydrolipoamide S-acetyltransferase (DLAT), leading to the aggregation of lip-DLAT and the loss of iron–sulfur cluster (Fe-S) proteins, subsequently inducing proteotoxic stress and ultimately cell death [5]. Cuproptosis invigorates research exploiting the unique role of copper to treat cancers, but its high dependence on intracellular Cu accumulation and mitochondrial respiration limits its application, especially for the cancer cells that rely on glycolysis for energy [5]. Therefore, it may be feasible to enhance susceptibility to cuproptosis via the integration of copperionophores and other substances to modify the metabolic pathways of cancer cells.

As a healthy beverage consumed worldwide, green tea is historically associated with enormous health benefits against multiple diseases, including cancer. The health effects of tea are largely attributed to polyphenols, especially EGCG [6]. The anticancer effects of EGCG and its mechanisms have received extensive attention. It is reported that EGCG promotes apoptosis and autophagy in oral cancer and has a synergistic effect with doxorubicin in Hep3B cells [7,8]. EGCG also increased the sensitivity of cancer cells to platinum drugs, which share a similar metabolic pathway to copper [9,10]. It was proven that the bioactivity of EGCG is altered in the presence of copper. Some researchers believe that EGCG and copper produce free radicals to cause oxidative stress, and others found that EGCG promotes an increase in intracellular copper levels [11,12], but whether the interaction between EGCG and copper can promote cuproptosis, especially in glycolytic-dependent cancer cells, is still unclear.

In this study, we examined the combined effect of EGCG and various copper ionophores on HCC using both cell culture and animal models, and the results showed that combining EGCG with copper ionophores resulted in a strong enhancement of efficacy against HCC in both the in vitro and in vivo models, which was attributed to increased cuproptosis. Furthermore, we found that EGCG can inhibit the expression of MTF1 to achieve the transcriptional repression of ATP7B, thereby reducing copper output and promoting cuproptosis. The findings provide a novel mechanistic support for EGCG to exert anticancer activity.

## 2. Materials and Methods

### 2.1. Reagents and Chemicals

(−)-Epigallocatechin gallate (EGCG, #HY-13653, 99.87% purity), Elesclomol (ES, #HY-12040), Disulfiram (DSF, #HY-B0240), Z-Vad-fmk (#HY-16658B), Ac-FLTD-cmk (#HY-111675), Ferrostatin-1 (Fer-1, #HY-100579), Deferoxamine (DFO, #HY-B1625), DPQ (#HY-114869), and MnTE-2-PyP chloride (MnPP, HY-130574) were purchased from MCE (Monmouth Junction, NJ, USA). CuCl_2_ (#C106775), FeCl_2_ (#I106504), FeCl_3_ (#I141414), CoCl_2_ (#C299372), NiCl_2_ (#N433838), AlCl_3_ (#A104930), 8-hydroxyquinoline (8-HQ, #H119327), and tetrathiomolybdate (TTM, #A189030) were purchased from Aladdin (Wuhan, China). Necrostatin-1 (Nec-1, #480065), Necrosulfonamide (NSA, #480073), Chloroquine phosphate (CQ, #C6628), Acetylcysteine (NAC, #1009005), and ZnCl_2_ (#703516) were purchased from Sigma-Aldrich (St. Louis, MO, USA). All reagents were dissolved following the manufacturers’ instructions. Catalase (CAT) was purchased from Solarbio, Beijing, China (C8070, U ≥ 2000 units/mg protein).

### 2.2. Cell Culture Studies

The HepG2 (ATCC, HB-8065) cell line was obtained from the American Tissue Culture Collection (ATCC, Manassas, VA, USA). The SMMC-7721 cell line was obtained from the Library of Tumor Cells of the Chinese Academy of Medical Sciences (Beijing, China). All the cells were cultured in a 37 °C incubator in a 5% CO_2_ atmosphere. HepG2 cells were cultured in Dulbecco’s modified Eagle medium (DMEM, Pricella, Suzhou, China), supplemented with 10% fetal bovine serum and 10,000 U/mL of penicillin–streptomycin. The SMMC-7721 cells were cultured in RPMI-1640 (Solarbio, China) medium supplemented with 10% fetal bovine serum and 10,000 U/mL penicillin–streptomycin.

### 2.3. Cell Death Assays

Cell death was measured using flow cytometry. Briefly, cells were seeded in 6-well plates and incubated overnight. After treatment, cells were trypsinized and collected. After washing with phosphate-buffered saline (PBS), the cells were resuspended in 300 μL PBS and stained with 5 μL 7-Aminoactinomycin D (7-AAD) and 5 μL Annexin V-APC (MBL, Tokyo, Japan, 20-6410-KIT) at room temperature for 20 min. The fraction of dead cells was measured using a BD Accuri C6 flow cytometer (BD Bioscience, Franklin Lakes, NJ, USA) and analyzed using FlowJo 10 software. All experiments were performed in triplicate.

### 2.4. Colony Formation Assay

The cells were grown in 6-well dishes with a cell density of 1000 cells per well. After the 14-day duration, the cells were immobilized with 4% paraformaldehyde and subjected to crystal violet staining. The ability of the cell colony to form was subsequently evaluated based on the presence of colonies.

### 2.5. Crystal Violet Staining

The cell viability was determined via crystal violet staining. Briefly, cells were seeded in 12-well plates and incubated overnight. After treatment, the cells were fixed with a 1% glutaraldehyde solution for 15 min and then stained with 0.02% crystal violet solution for 30 min. After washing and air-drying, the cells were soluble in 75% ethanol. The absorbance at 590 nm was measured using a plate reader (Thermo, Multiskan FC, Waltham, MA, USA).

### 2.6. Western Blotting Analysis

Western blotting was performed as previously described. Briefly, cells were lysed in an ice-cold radio immunoprecipitation assay lysis (RIPA) buffer (Solarbio, #R0010) containing protease inhibitors. The protein concentration of lysates was quantified using a bicinchoninic acid (BCA) protein assay kit from Solarbio (#XYW-3). Equal amounts of protein were loaded onto sodium dodecyl sulfate (SDS)–polyacrylamide gel electrophoresis and transferred onto a nitrocellulose membrane (Millipore, Billerica, MA, USA) after separation. The membrane was blocked with 5% non-fat milk at room temperature for 1 h, followed by incubation with the primary antibody at 4 °C overnight. After incubation with the corresponding secondary antibody (MBL), the target protein was visualized by chemiluminescence. The primary antibodies and concentrations used for Western blotting were HSP70 (abmat, Shanghai, China, #M20033), DLAT (ABclonal, Wuhan, China, #A14530), ATP7B (proteintech, Shanghai, China, 19786-1-AP; abmart, Shanghai, China, T58616), CTR1 (abmart, T510261F), MTF1 (abmart, MG775483), beta-actin (ABclonal, #AC004), and GAPDH (ABclonal, #A19056).

### 2.7. Immunofluorescence (IF)

For IF staining, cells in confocal dishes (BeyoGold™, Shanghai, China, #FCFC020) were fixed with 4% paraformaldehyde (Solarbio, P1110) for 15 min and incubated with 0.3% Triton-X-100 (Solarbio, T8200) for 10 m min at room temperature. Then, the cells were blocked by 10% bovine serum albumin (BSA, Solarbio, A8010) in PBS for 1 h, and incubated with primary antibodies at 4 °C overnight. After PBS washing three times, cells were bound with fluorescence-conjugated secondary antibodies (MBL, 238; Proteintech, SA00013-2) for 1 h and nuclei were stained with DAPI (Solarbio, # S2110). Images were captured with the confocal microscope (Nikon, Tokyo, Japan).

### 2.8. Flow Cytometric Analysis of the Cellular ROS

After administration, the cultured cells were added into a serum-free medium containing 2′,7′-dichlorofluorescein (DCF) fluorescent dye (Thermo Fisher, USA, #D399) and incubated at 37 °C for 30 min. The excess dye was washed off using precooled PBS solution, and the cells were collected. The serum-free medium was stored in an ice tank for immediate detection via flow cytometry (BD Bioscience, USA). (Excitation wavelength: 488 nm; Emission wavelength: 530 nm.).

### 2.9. Determination of Copper Content in Cells

The intensity of copper fluorescence in cells was observed using coppersensor 1 (CS1, MCE, #HY-141511). Briefly, the HepG2 cells in the logarithmic growth phase were inoculated in a six-well plate and incubated at 37 °C in a 5% CO_2_ atmosphere overnight. After treatment, the cells were washed with PBS, incubated with 5 μM CS1 for 20 min at 37 °C, and the nuclei were stained with DAPI. Images were captured with the microscope (Nikon).

The copper content in the cells was determined using inductively coupled plasma mass spectrometry (ICP-MS). Briefly, the cell was collected and digested with 0.1 mL of nitric acid at 65 °C for one hour, and then diluted with 0.1 mL of water. Partial cell suspension was removed for protein quantification. The copper content of the cell sample was measured using Perkin Elmer NexION 300D ICP-MS (Perkin Elmer, Norwalk, CT, USA) and normalized to the protein concentration.

### 2.10. Reverse Transcription and Quantitative Real-Time Polymerase Chain Reaction

Total RNA was extracted from cell samples and purified using the RNApure Fast Tissue&Cell Kit (CWBIO, Taizhou, China, 0599S). After reverse transcription with the cDNA Synthesis Mix (CWBIO, CW2582W), the mRNA expression levels were determined by a quantitative real-time PCR system (CWBIO, CW0957S), and each gene was normalized to beta-actin.

In the experiments, the following primer sequences were used:

ATP7B, Forward, 5′-CTTGGGATACTGCACGGACTTC-3′, and Reverse 5′-CCTCAGCCACTCACGGTTTC-3′;

CTR1, Forward, 5′-TTGGCTTTAAGAATGTGGACCT-3′, and Reverse 5′-GACTTGTGACTTACGCAGCA-3′;

Beta-actin, Forward, 5′-TGTGGCATCCACGAAACTAC-3′, and Reverse 5′-GGAGCAATGATCTTGATCTTCA-3′.

### 2.11. Detection of Intracellular GSH

The commercially available GSH assay kit (Nanjing Jiancheng, Nanjing, China, A006-2-1) was used to detect the depletion of GSH. After incubation, HepG2 cells were collected, suspended in 0.2 mL PBS, and then processed by an ultrasound cell crusher. Then, 100 μL of the above cells was added to a plate, and the regents were added sequentially according to the instructions. After incubation at room temperature for 5 min, the absorbance was measured at 405 nm.

### 2.12. Cu-ATPase Activity Measurement

HepG2 cells were seeded in 6-well plates and incubated with EGCG and ES + CuCl_2_ for 24 h. Then, the cells were collected and washed with normal saline three times. According to the instructions of the Cu-ATPase assay kit (Nanjing Jiancheng, A057-1-1), the reagent was added successively for the reaction, and then the activity of Cu-ATPase was calculated according to the content of inorganic phosphorus produced.

### 2.13. Mice Xenograft Model

Male Balb/c nude mice (5–6 weeks of age) were obtained from Beijing Vital River Laboratory Animal Technology Company and housed in the laboratory-animal research center of China Agricultural University. HepG2 cells (5 × 10^6^) were resuspended with Matrigel (1:1, *v*/*v*) (Corning, Bedford, MA, USA), which was injected subcutaneously into the right flank of each mouse. When implanted tumors reached 100 mm^3^, mice were randomly divided into 4 groups (n = 6) and then intraperitoneally injected with the following every day for three weeks: solvent, EGCG (10 mg/kg), elesclomol (10 mg/kg), EGCG (10 mg/kg) + elesclomol (10 mg/kg). Tumors were measured with a caliper and tumor volumes were calculated using the following formula: length × width × width × 0.5. At the termination of the experiments, the xenografts from the euthanized mice were photographed and weighed. The animal care and procedures were approved by the China Agricultural University Institutional Animal Care and Use Committee (No. AW42604202-4-7).

### 2.14. Statistics and Reproducibility

The results of the cell culture experiments were collected and analyzed from at least three independent repeats. Volumes or photon counts from at least six tumors or mice in each group were plotted. For immunoblots, the experiments have been repeated at least twice with similar results, and the representative data were shown. No statistical method was used to pre-determine the sample size. The data are presented as means ± standard deviations (SD). Statistical comparisons were performed by one-way ANOVA followed by Tukey’s post hoc test using GraphPad Prism 8.4.3. A *p* value < 0.05 was considered significant. Graphs were drawn using GraphPad Prism (version 8.4.3 for Windows).

## 3. Results

### 3.1. EGCG and Copper Ionophore Combinational Treatment Promotes Cell Death

**Figure 1:**
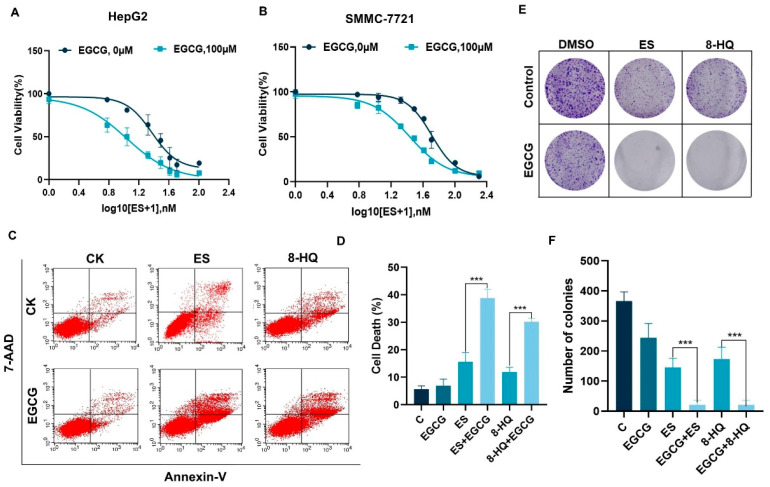
ECGG enhanced copper ionophore-mediated hepatocellular carcinoma cell death in vitro. (**A**,**B**) Cell viability of HepG2 (**A**) and SMMC-7721 (**B**) after gradient concentrations of ES 24 h treatment with DMSO or 100 μM EGCG was measured with crystal violet staining. (**C**,**D**) The cell death rate and quantification of 100 μM EGCG combined with 30 nM ES or 3 μM 8-HQ treatment of HepG2 and SMMC-7721 cells for 24 h using flow cytometry. (**E**,**F**) Colony formation after treatment with 50 μM EGCG combined with 20 nM ES or 2 μM 8-HQ in HepG2. For (**A**–**F**), media were supplemented with 2 μM CuCl_2_. (The data are presented as the mean ± standard deviation. n = 3, *** *p* < 0.001.).

Copper ionophores possess the ability to bind to copper ions and transport them into cells, thereby serving as valuable instruments for the investigation of copper toxicity [13]. To investigate the influence of EGCG on copper-induced cell death, we firstly treated HepG2 and SMMC-7721 cells with EGCG and gradient concentrations of ES. The results showed that EGCG sensitized the inhibition of the cell viability of HCC cells induced by ES and copper ion premixture. (Figure 1A,B). To exclude the specificity of ES, additional copper ionophores, 8-HQ, and DSF were adopted, and enhancing the effect of EGCG on the reduction in cell survival rate was also induced by its combination with 8-HQ or DSF (Figure S1A–D). The cell death rate was detected using annexin v/7-AAD staining, and it was found that 100 μM EGCG did not exhibit a significant effect on cell death, while the cell mortality rate following combined treatment was observed to be twice that of the treatments with ES or 8-HQ treatment alone (Figure 1C,D), indicating that combined treatment could markedly augment the induction of cell death. The decrease in the quantity of tumor clones was significantly more pronounced in the group receiving combination therapy than in the group undergoing single treatment (Figure 1E,F). In summary, EGCG enhanced copper-induced cell death in liver cancer cells.

### 3.2. EGCG and Copper Ionophore Combinational Treatment Induces Cuproptosis

**Figure 2:**
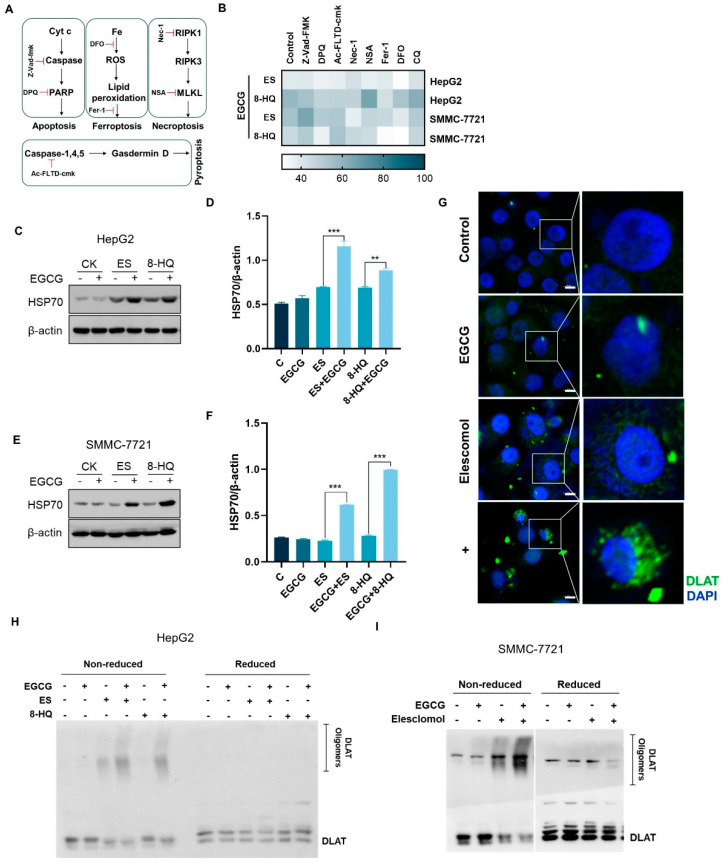
EGCG combined with copper ionophores promotes hepatocellular carcinoma cell cuproptosis. (**A**) Diagram of apoptosis, necroptosis, ferroptosis, and pyroptosis mechanisms. (**B**) Heatmap of cell viability after 100 μM EGCG combined with 30 nM ES or 3 μM 8-HQ treatment for 24 h with 20 μM Z-Vad-fmk, 20 μM DPQ, 10 μM Ac-FLTD-cmk, 10 μM Nec-1, 10 μM NSA, 10 μM Fer-1, 10 μM DFO, and 10 μM autophagy inhibitor CQ. (**C**–**F**) Expression of HSP70 in liver cancer cells treated with 100 μM EGCG combined with 30 nM ES and 3 μM 8-HQ for 24 h. (**G**) DLAT protein aggregation was analyzed using immunofluorescence (green, DLAT; blue, DAPI). White scale bars on full tiles are 10 μm. (**H**,**I**) Western blotting detection of DLAT protein aggregation in liver cancer cells. For (**B**–**I**), media were supplemented with 2 μM CuCl_2_. (The data are presented as the mean ± standard deviation. n = 3, ** *p* < 0.01, and *** *p* < 0.001.).

To investigate the type of cell death elicited by the combination of EGCG and copper ionophores, cells were subjected to various cell death inhibitors (Figure 2A). As shown in Figure 2B, all inhibitors tested were ineffective in preventing the cell death induced by the combined treatment, ruling out the involvement of apoptosis, necroptosis, pyroptosis, or ferroptosis in EGCG and copper ionophore combination-induced cytotoxicity [5,14,15,16,17].

Previous reports have demonstrated that ES can induce ROS-dependent cell death [18]. To investigate whether the combined treatment induces cell death through ROS accumulation, the DCF probe was employed to detect ROS in the cells post treatment. The results indicated that both ES and the combined treatment had a certain degree of increasing ROS generation, but there was no significant difference between them (Figure S2A,B). Additionally, the cells were pre-treated with free radical scavengers, NAC, MnPP, and CAT, and the results showed that the elimination of ROS did not prevent cell death (Figure S2C,D). Additionally, since copper ions facilitate the oxidation of EGCG, we investigated the combined effects of EGCG and CuCl_2_ in the absence of ES, revealing no significant alterations with EGCG alone (Figure S2E). These results suggest that elevated copper levels within the cells may lead to ROS production, but that ROS may not play a critical role in the cell death induced by copper or the combined treatment.

Cuproptosis refers to proteotoxic stress death caused by the aggregation of lipoylated proteins, a process significantly influenced by copper [5]. We found that the application of copper chelator TTM markedly reduced combination-induced cell death (Figure S3A,B). To exclude the potential confounding effects of other metal ions, we introduced other metals during the culture process. However, none of these metals exacerbated cell death (Figure S3C). Glutathione (GSH) serves as a copper chelator, and the introduction of exogenous GSH was found to inhibit cell death (Figure S3D). Conversely, the depletion of endogenous GSH through the glutamate cysteine synthase inhibitor BSO reduced cell viability (Figure S3D). These results suggest that copper is indispensable for the mechanism by which EGCG enhanced the sensitivity of copper ionophores.

Proteotoxic stress was identified as the primary factor leading to cuproptosis [5]. Heat Shock Protein 70 (HSP70), as a marker of acute proteotoxic stress, significantly increased in the combined group (Figure 2C–F). In addition, the aggregation of lipoylated proteins, such as DLAT, is also an important sign of cuproptosis. Thus, immunofluorescence experiments were carried out to visualize the distribution of DLAT in liver cancer cells. Co-treatment of EGCG and ES significantly augmented DLAT puncta in HepG2 cells compared to ES alone (Figure 2G). Western blotting results further verified this conclusion (Figure 2H,I). Taken together, these results indicated that EGCG promoted copper-induced cuproptosis in liver cancer cells.

### 3.3. EGCG Increases Intracellular Copper Accumulation by Down-Regulating ATP7B Expression

**Figure 3:**
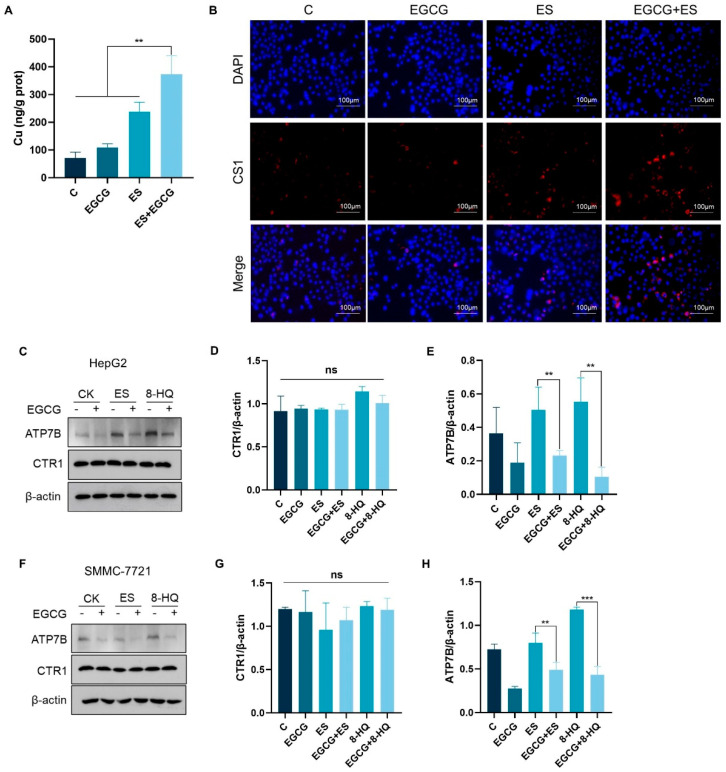
EGCG inhibited ATP7B expression and increased intracellular copper accumulation. (**A**) Copper levels were assessed via ICP-MS in HepG2 cells treated with 30 nM ES with or without 100 μM EGCG for 18 h (n = 3). (**B**) Representative images of copper fluorescence in HepG2 cells treated with or without drugs for 18 h (red, copper; blue, DAPI). White scale bars on full tiled images are 100 μm. (**C**–**H**) ATP7B and CTR1 protein expression in HepG2 and SMMC-7721 cells treated with or without drugs for 18 h (n= 3). For A-H, media were supplemented with 2 μM CuCl_2_. The data are presented as the mean ± standard deviation. ** *p* < 0.01, *** *p* < 0.001, and ns indicates no significant difference.

To elucidate the mechanism by which EGCG facilitated cuproptosis, we first investigated alterations in intracellular copper concentrations. The results of ICP-MS showed that treatment with ES alone for 24 h led to a modest increase in intracellular copper levels, while the group receiving combined treatment exhibited a significantly greater elevation in copper levels compared to each agent treatment alone (Figure 3A). Furthermore, compound CS1, which can interact with intracellular copper to emit fluorescence, was used as an indicator of copper to verify the changes in copper levels. Fluorescence microscopy observations revealed a pronounced increase in the copper ion concentration in cells subjected to combined treatment (Figure 3B). An increase in the copper concentration is supposed to reduce the GSH content in cells [19], and as expected, our results showed a reduction in the GSH levels after co-treatment for 24 h (Figure S4A), further supporting that EGCG can promote intracellular copper accumulation.

Intracellular copper levels are meticulously regulated by copper metabolism mechanisms. To elucidate the mechanism through which EGCG facilitated copper accumulation, we assessed the expression levels of proteins related to cellular copper metabolism. The results showed that the expression of the copper uptake protein copper transporter 1 (CTR1) remained relatively stable throughout the treatment period, while the expression and enzyme activity of the copper efflux protein ATP7B decreased significantly following EGCG treatment (Figure 3C–H and Figure S4B). ATP7B is typically localized in the trans-Golgi network (TGN) under normal copper level status. In response to elevated copper concentrations, ATP7B will translocate to vesicles and subsequently fuse with the plasma membrane. Following this translocation, ATP7B facilitates the efflux of copper from the cells [20]. Localization analysis of ATP7B revealed that, after combined treatment, the protein predominantly localized to the cell membrane, which was consistent with the excessively elevated intracellular copper levels, and that EGCG did not affect ATP7B localization (Figure S5). In summary, EGCG induces the accumulation of copper in liver cancer cells by lowering the expression of ATP7B.

### 3.4. EGCG Regulates ATP7B Transcription by Inhibiting MTF1 Expression

**Figure 4:**
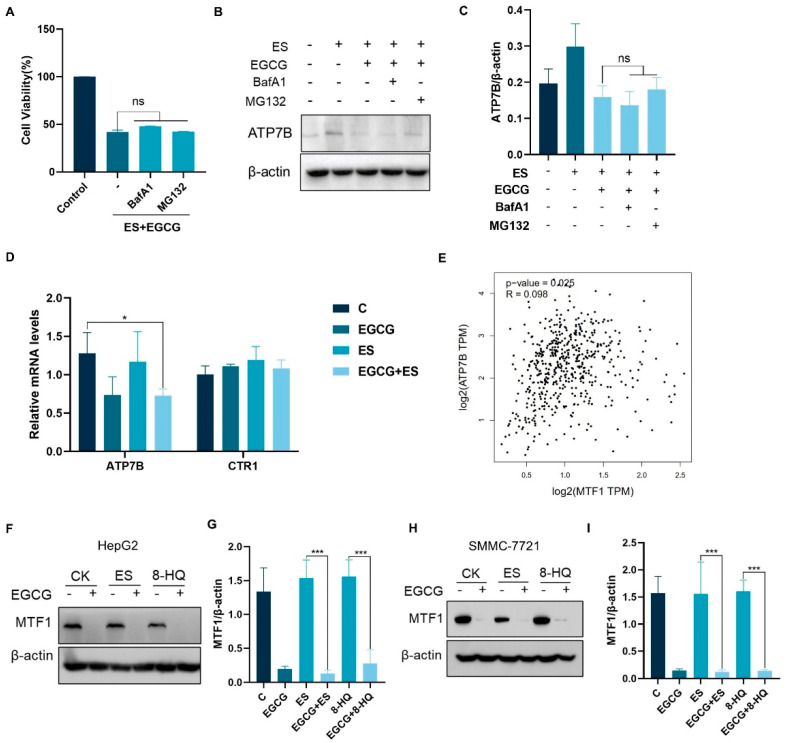
EGCG regulates ATP7B transcription through MTF1. (**A**–**C**) Cell viability (n = 3) (**A**), ATP7B expression (**B**), and quantification (n = 3) (**C**) after pretreatment with 50 nM BafA1 and 10 μM MG132 for 6 h followed by drug treatment for 18 h. (**D**) Relative mRNA levels of genes of CTR1 and ATP7B in HepG2 cells after 12 h of drug treatment (n = 3). (**E**) A correlation analysis was performed to evaluate the association between MTF1 and ATP7B using GEPIA2, (**F–I**) MTF1 expression levels in HCC cells treated with drugs after 12 h. The concentration of EGCG was 100 μM, ES was 30 nM, and 8-HQ was 3 μM. For (**A**–**I**), media were supplemented with 2 μM CuCl_2_. (The data are presented as the mean ± standard deviation. * *p* < 0.05, *** *p* < 0.001, and ns indicates no significant difference.).

The expression of ATP7B is subject to regulation at various levels, including transcription and post translation. To explore the mechanisms underlying the down-regulation of ATP7B by EGCG, we first examined whether EGCG induced degradation of the ATP7B protein. Protein degradation can occur through autophagic mechanisms or via the ubiquitin–proteasome pathway, both of which can be inhibited using specific inhibitors to potentially restore the levels of degraded proteins. Cells were pre-treated with autophagy inhibitor BafA1, as well as the proteasome inhibitor MG132, to impede protein degradation. Under such conditions, the synergistic cytotoxic effects were not mitigated and the reduction in ATP7B expression by EGCG was not affected (Figure 4A–C). These results suggest that the down-regulation of ATP7B protein expression by EGCG was not attributable to its ability to promote ATP7B degradation.

We next questioned whether EGCG suppressed ATP7B transcription. qPCR was employed to evaluate the expression levels of mRNA associated with copper metabolism. The results showed that the transcription of ATP7B decreased significantly following treatment with EGCG, indicating that EGCG might affect the transcriptional process of ATP7B (Figure 4D). It has been reported that MTF1, a classic metal sensing transcription factor, is capable of regulating metal homeostasis [21]. According to The Cancer Genome Atlas Program—the liver hepatocellular carcinoma (TCGA-LIHC) dataset, ATP7B mRNA expression, and MTF1 showed a clear positive correlation (Figure 4E). The analysis of MTF1 expression showed that treatment with EGCG could also significantly down-regulate the expression of MTF1 (Figure 4F,G). The results suggest that EGCG may reduce ATP7B transcription by down-regulating MTF1.

### 3.5. Combination of EGCG and ES Exhibits Enhanced Anti-Hepatocellular Carcinoma Effects In Vivo

**Figure 5:**
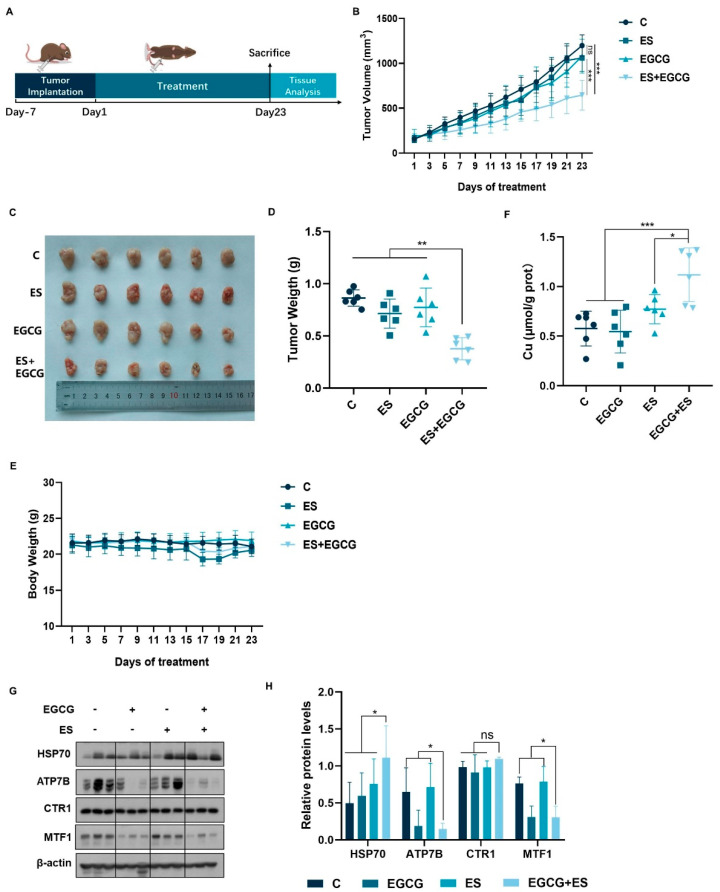
ECGG promoted cuproptosis in liver cancer in vivo. (**A**) Establishment of a xenograft model in nude mice and an experimental schematic diagram (n = 6). (**B**) Tumor growth curve. (**C**) Photographs of tumors. (**D**) Tumor weight. (**E**) Body weight of mice. (**F**) Tumor copper levels. (**G,H**) Protein content of tumor tissues. (The data are expressed as mean ± standard deviation, * *p* < 0.05, ** *p* < 0.01, *** *p* < 0.001 and ns indicates no significant difference.).

Based on the results of in vitro experiments, HepG2 cells were used to establish a subcutaneous liver cancer xenotransplantation model in mice to further investigate the combined effects of ES and EGCG in vivo. The experimental flowchart is illustrated in Figure 5A. The tumor volumes (Figure 5B) and weights (Figure 5D) of the four groups were compared. As expected, single administration of the drugs at the experimental dose had no significant inhibitory effect on tumor growth, while the combined group notably impeded tumor progression (Figure 5B–D). Moreover, no overt weight loss was observed in single or combined administration of the drugs at the experimental dose (Figure 5E).

To investigate whether EGCG facilitated cuproptosis and its impact on copper levels in tumor cells in the subcutaneous tumor model of mice, we assessed the copper concentration, the expression of copper metabolism-related proteins, and HSP70 proteins in tumor tissues. Treatment with ES alone increased the copper levels in tumor tissues, whereas EGCG treatment alone did not produce a similar effect. However, the combined treatment resulted in a more pronounced increase in copper levels (Figure 5F). Additionally, the expression of HSP70 was elevated in the combined treatment group, indicating that EGCG may also promote the occurrence of cuproptosis in vivo (Figure 5G,H). Furthermore, EGCG also down-regulated the expression of ATP7B and MTF1 in tumor tissues without affecting CTR1 expression (Figure 5G,H). Taken together, the xenograft studies confirmed that EGCG promoted cuproptosis in liver cancer in vivo.

## 4. Discussion

The discovery of cuproptosis offers a new option for cancer treatment. However, cancer cells are generally cuproptosis-reluctant, due to their preference for glycolytic metabolism. The agents that can sensitize cancer cells to copper-mediated cuproptosis are clearly needed for developing cuproptosis-based therapeutic regimes. To this end, we addressed the sensitizing effect of EGCG on copper-induced cuproptosis in liver cancer cells using both in vitro and in vivo models. The results showed that EGCG greatly potentiated liver cancer cells to copper-induced cuproptosis, which in turn contributed to the improved efficacy against HCC. The findings imply that EGCG holds promising potential to be developed as a novel sensitizer for cuproptosis.

EGCG is a prevalent polyphenol found in tea, recognized for its diverse anticancer properties. Research has demonstrated that EGCG, whether administered independently or in conjunction with other pharmacological agents, possesses the capability to suppress the proliferation of HCC cell lines [22,23,24]. In this study, copper ionophores ES, 8-HQ, and DSF were used. The results showed that the combination of EGCG and copper ionophores significantly increased the mortality rate of HCC cells and up-regulated the levels of HSP70 and oligomerization of DLAT, indicating that EGCG facilitated the incidence of cuproptosis in HCC cells.

Currently, numerous anticancer mechanisms associated with EGCG have been proposed. Some researchers posit that the oxidation of EGCG leads to the generation of a substantial quantity of free radicals, which in turn induces oxidative damage and apoptosis [25]. Transition metal ions, particularly copper ions, are known to facilitate the oxidation of EGCG, resulting in the production of hydroxyl radicals and hydrogen peroxide, thereby potentially enhancing the anticancer efficacy of EGCG [11]. However, in the present study, we assessed the cell survival rate following the combination of EGCG and copper ionophores, while employing inhibitors such as NAC, MnPP, and catalase to eliminate ROS both intracellularly and extracellularly. Our findings revealed no significant differences in cell survival, indicating that ROS may not play a critical role in this context. The transcription factor nuclear factor erythroid 2-related factor 2 (Nrf2) is known to regulate the expression of various antioxidant and detoxification enzymes. Previous studies have demonstrated that A549 cells can up-regulate the expression of heme oxygenase-1 (HO-1) and its transcription factor Nrf2 in vitro, thereby enhancing resistance to apoptosis induced by EGCG [26]. Furthermore, Nrf2 and its target genes are up-regulated in the liver of mice administered with EGCG intraperitoneally, promoting an adaptive response in hepatic tissues [27]. Consequently, we hypothesize that the lack of involvement of ROS may be attributed to the inhibitory effect of Nrf2, which functions as an antioxidant defense mechanism within cells, thereby mitigating the oxidation of EGCG.

EGCG can target copper accumulation and induce DNA fragmentation in DEN-induced rat hepatocellular carcinoma, which can be inhibited by the cell membrane-permeable copper chelator neocuproine [12]. It is suggested that the interaction between EGCG and copper may hold therapeutic potential in cancer treatment, but the specific mechanism remains to be elucidated. Results from ICP-MS and CS1 fluorescence detection showed a marked increase in intracellular copper levels within the combination-treated group, indicating that EGCG may promote cuproptosis via intracellular copper accumulation.

Cellular copper levels are meticulously regulated by the copper metabolism network. Copper enters cells via CTR1 proteins and then binds to cytosolic copper chaperones for delivery to specific subcellular regions [4]. With elevated intracellular copper, ATP7B and ATP7A translocate from the TGN to the cell periphery to expel excess copper [28]. To investigate the regulatory effects of EGCG on intracellular copper levels, we examined the expression of proteins associated with copper metabolism and found that ATP7B expression was significantly decreased following EGCG treatment. ATP7B is a P-type ATPase and is highly expressed in liver, which is responsible for pumping copper at the expense of ATP hydrolysis. Mutations in the ATP7B gene may lead to the impaired localization of its protein product and cause toxic copper accumulation in the liver [29,30]. The expression of ATP7B is regulated by various proteins, including MTF1.

As a transcription factor, MTF1 will transfer from the cytosol to the nucleus when heavy metals accumulate and bind to MRE in the promoters of target genes, driving the expression of metallothionein, metal transporters, and other metal-induced proteins [31,32]. In drosophila, MTF1 promotes the expression of DmATP7 protein in response to copper accumulation, which is homologous to the mammalian ATP7B protein [33]. The human ATP7B promoter contains four metal response elements (MREs): MREa, MREe, MREc, and MREd. Studies have shown that MTF1 can drive the ATP7B promoter by binding to MREe in the ATP7B promoter [21]. A mutation in the ATP7B promoter region of a patient with Wilson’s disease disrupted MTF1 binding at this site, leading to the inadequate transcription of ATP7B [34]. These findings proved that MTF1 was involved in the transcription and expression of the ATP7B gene. Additionally, MTF1 was also confirmed to be an anti-cuproptosis gene, with its knockout promoting the occurrence of cuproptosis [5]. In our study, the down-regulation of ATP7B expression following EGCG treatment was not reversed by a autophagy inhibitor or proteasome inhibitor, suggesting that it may not be due to increased degradation. In addition, the mRNA analysis indicated that EGCG may inhibit ATP7B transcription. Consequently, we speculate that EGCG promotes cuproptosis by interfering with the MTF1/ATP7B axis, although further investigation is required to elucidate the mechanisms by which EGCG downregulates MTF1 expression.

In addition, our study found that EGCG down-regulated the expression of MTF1 and ATP7B, but was not affected by copper, that is, EGCG treatment alone was capable of down-regulating the expression of ATP7B without causing cuproptosis in cells or tumor tissues. This observation implies that, in the absence of copper ionophores, cells predominantly depend on CTR1 for copper uptake. Even under conditions of an elevated copper concentration in the culture medium, cells do not actively absorb excessive amounts of copper. When copper is introduced directly into the cells via copper ionophores, the down-regulation of ATP7B disrupts the copper export mechanism, leading to the progressive accumulation of copper and exacerbating the incidence of cuproptosis.

This paper presents several limitations. Firstly, while the oral administration of EGCG is a common method for the human consumption of tea or EGCG products, the absorption and bioavailability of orally ingested EGCG in both animals and humans are notably low, as a significant portion of the consumed EGCG is metabolized by the intestinal microbiota. The intraperitoneal injection method employed in this study does not accurately reflect the typical mode of EGCG intake in humans. Future research could focus on strategies to enhance the efficacy of oral administration, such as modifying EGCG or creating a metal-polyphenol complex with copper to improve absorption efficiency and targeting [35]. Secondly, this study exclusively addresses the effects of EGCG on liver cancer, lacking an examination of its impact on other cancer types, which raises questions about its potential broad-spectrum efficacy. High-throughput screening across various tumor types could be conducted to identify those with the most favorable response for further investigation. Additionally, MTF1, a metal ion regulatory protein, warrants further exploration regarding the regulatory mechanisms and applications of EGCG in relation to MTF1.

## 5. Conclusions

In summary, we first report a new antitumor mechanism of EGCG, which disrupted intracellular copper homeostasis and facilitated cuproptosis in liver cancer cells by regulating the MTF1/ATP7B axis. These findings not only identify a new target of EGCG, but also provide a theoretical reference for its clinical application in the treatment of HCC.

## Data Availability

The datasets used and analyzed during the current study are available from the corresponding author upon request.

## Supplementary Materials

The following supporting information can be downloaded at: https://www.mdpi.com/article/10.3390/cells14060391/s1. Figure S1: EGCG enhanced copper ionophore-induced cell toxicity; Figure S2: EGCG promoting copper-induced death is unrelated to ROS; Figure S3: Copper plays an important role in EGCG-induced synergism; and Figure S4: EGCG increases intracellular copper accumulation. Figure S5: The subcellular localization of ATP7B.

## Abbreviations

7-AAD	7-Aminoactinomycin D
CAT	Catalase
HCC	Hepatocellular carcinoma
DLAT	Dihydrolipoamide S-acetyltransferase
Fe-S cluster	Iron–sulfur cluster
EGCG	Epigallocatechin gallate
MTF1	Metal regulatory transcription factor
ATP7B	Copper-transporting P-type ATPase
ES	Elesclomol
8-HQ	8-Hydroxyquinoline
DSF	Disulfiram
ROS	Reactive oxygen species
DCF	2′,7′-dichlorofluorescein
NAC	N-acetylcysteine
TTM	Tetrathiomolybdate
GSH	Glutathione
BSO	Buthionine sulfoximine
HSP70	Heat Shock Protein 70
IF	Immunofluorescence
CTR1	Copper transporter 1
TGN	Trans-Golgi network
CQ	Chloroquine
BafA1	Bafilomycin A1
FINs	Class I ferroptosis inducers
MBDs	Metal binding domains
MREs	Metal response elements
CS1	Coppersensor 1
TCGA	The Cancer Genome Atlas Program
LIHC	Liver hepatocellular carcinoma
Nrf2	Nuclear factor erythroid 2-related factor 2
HO-1	Heme oxygenase-1
ICP-MS	Inductively coupled plasma mass
