# Supplementary material for: Epigallocatechin Gallate Promotes Cuproptosis via the MTF1/ATP7B Axis in Hepatocellular Carcinoma
Source: Cells. 2025 Mar 7;14(6):391. doi: 10.3390/cells14060391 (PMC11941326; doi:10.3390/cells14060391)
Supplement: Supplementary file 1 [file cells-14-00391-s001.zip › cells-3485764-supplementary.pdf]

Supplementary information for

Epigallocatechin Gallate promotes cuproptosis via MTF1/ATP7B axis in hepatocellular carcinoma

Yuhan Fu, Lirui Hou, Kai Han, Chong Zhao, Hongbo Hu* and Shutao Yin*

Corresponding author: Shutao Yin (yinshutao@cau.edu.cn); Hongbo Hu, (hongbo@cau.edu.cn)

This file includes:

Figure S1
Figure S2
Figure S3
Figure S4
Figure S5

Figure S1

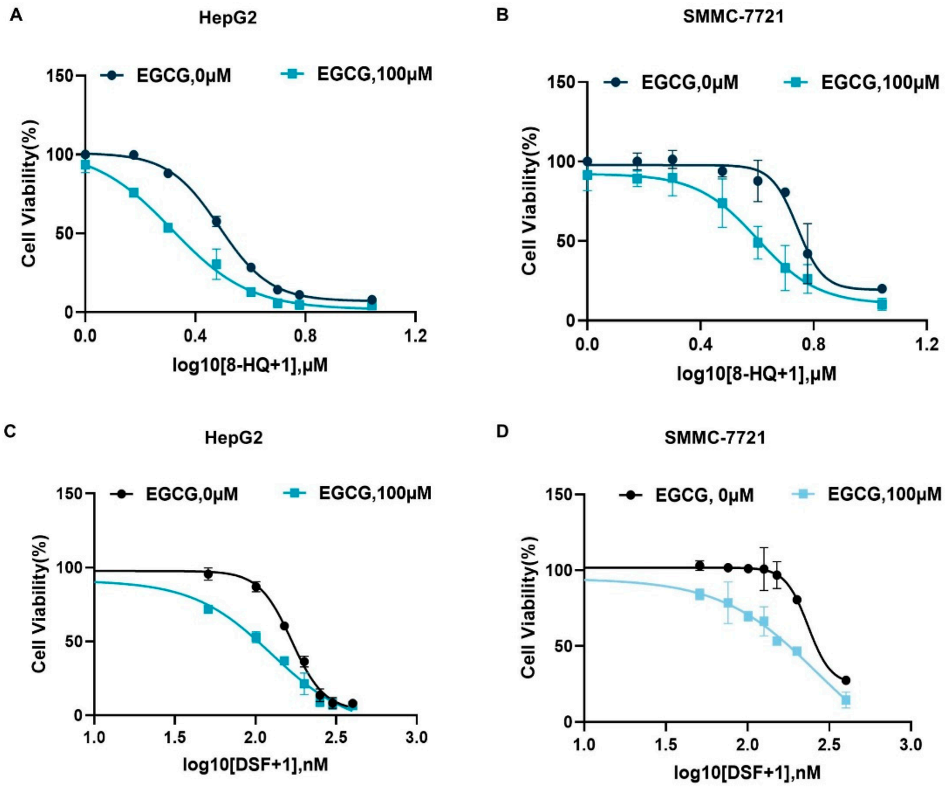

Figure S1. EGCG enhanced copper ionophores-induced cell toxicity. (A-B) Cell viability of HepG2 (A) and SMMC-7721 (B) after gradient concentrations of 8-HQ 24 h treatment with DMSO or 100 μM EGCG was measured with Crystal violet staining. (C-D) Cell viability of HepG2 (C) and SMMC-7721 (D) after gradient concentrations of DSF 24 h treatment with DMSO or 100 μM EGCG was measured with Crystal violet staining. For A-D, media were supplemented with 2 μM $CuCl_2$. (n=3, *$p<0.05$, **$p<0.01$, ***$p<0.001$.)

Figure S2

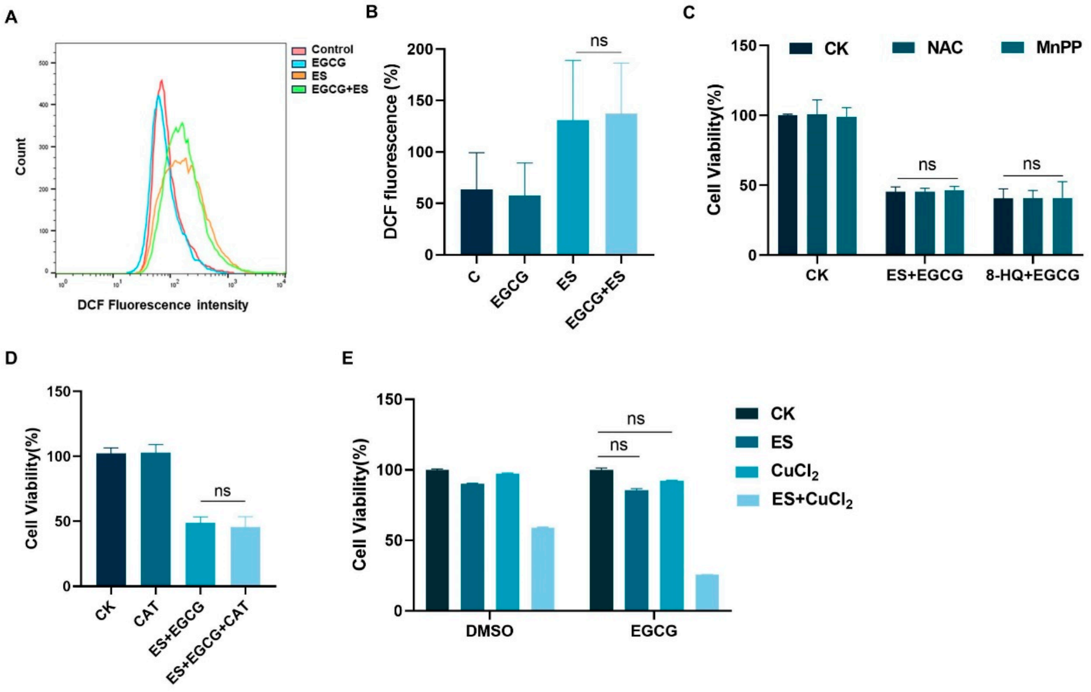

Figure S2. EGCG promoted copper-induced death is unrelated to ROS. (A-B) DCF probe was used to detect the ROS level in the single group and combined group，among which the concentration of EGCG was 100 μM and ES was 30 nM . (C) The survival rate of cells pretreated with 3 mM NAC or 10 μM MnPP for 3 h was detected after single and combined treatment. (D) The survival rate of cells treated with 30 nM ES, 100 μM EGCG and 1 mg/ml CAT for 24 h. (E) The survival rate of cells treated with 30 nM ES, 100 μM EGCG or 2 μM $CuCl_2$. For A-D, media were supplemented with 2 μM $CuCl_2$. (n=3, **p*<0.05, ***p*<0.01, ****p*<0.001, ns indicates no significant difference).

Figure S3

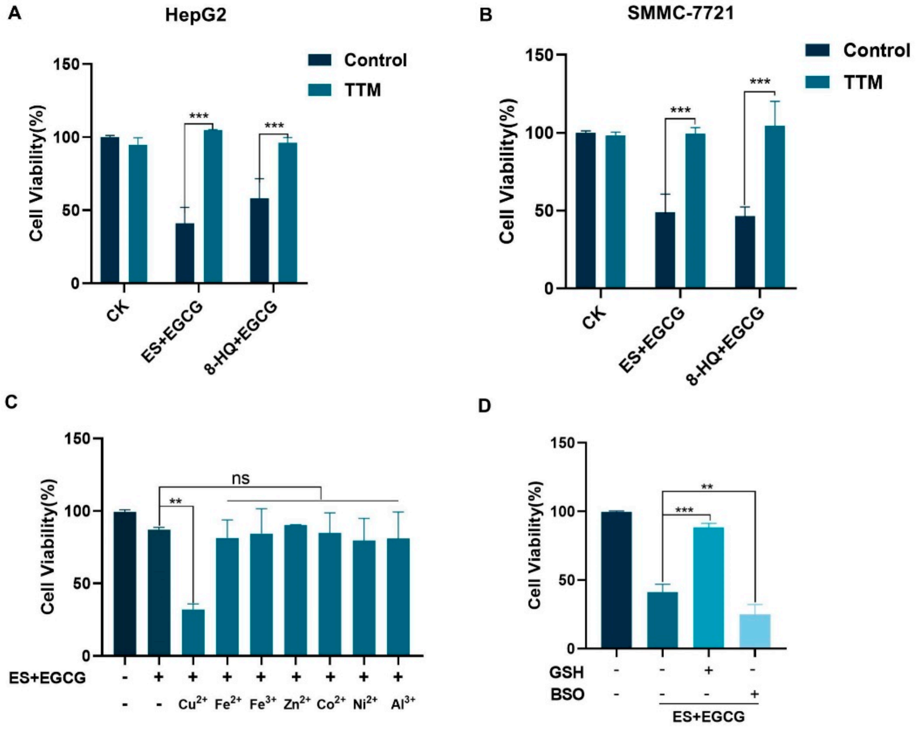

Figure S3. Copper plays an important role in EGCG-induced synergism. (A-B) Cell viability of HepG2 cells (A) and SMMC-7721 (B) after combined treatment with or without 20 μM copper chelator TTM for 24 h, among which the concentration of ES was 30 nM, 8-HQ was 3 μM， and EGCG was 100 μM . (C) Cell viability of HepG2 cells treated with 30 nM ES+100 μM EGCG and 2 μM $CuCl_2$, $FeCl_3$, $FeCl_2$, $ZnSO_4$, $CoCl_2$, $NiCl_2$, $AlCl_3$ for 24h, respectively. (D) Cell viability of HepG2 cells pretreated with 1 mM GSH, 10 μM BSO for 6h and following treated with 100 μM EGCG+30 nM ES for 24 h. For A and C, media were supplemented with 2 μM $CuCl_2$. (n=3, **p*<0.05, ***p*<0.01, ****p*<0.001, ns indicates no significant difference)

Figure S4

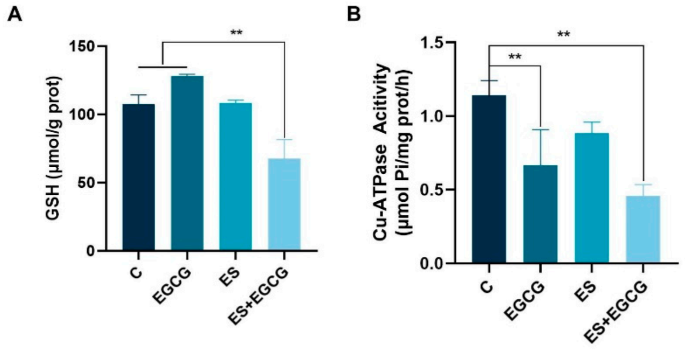

Figure S4. EGCG increases intracellular copper accumulation. (A) GSH content in liver cancer cells. (B) Cu-ATPase enzyme activity. The concentration of EGCG was 100 μM, ES was 30 nM and the processing time is 24 h. For A-B, media were supplemented with 2 μM $CuCl_2$. (n=3, **p*<0.05, ***p*<0.01, ****p*<0.001)

Figure S5

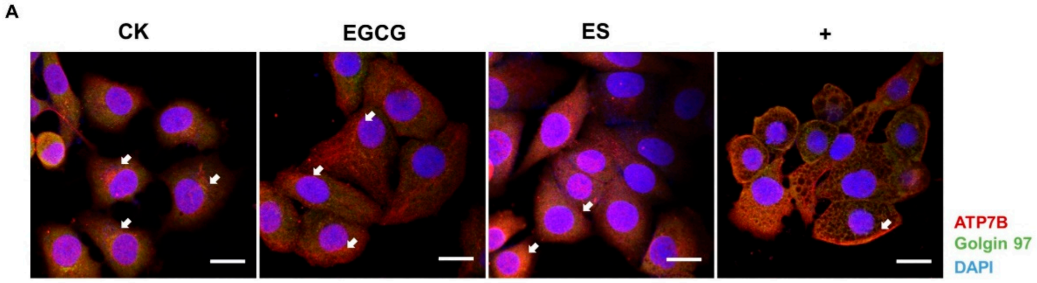

Figure S5. The subcellular localization of ATP7B. Fluorescence intensity and localization of ATP7B after 18h of 100 μM EGCG and 30 nM ES (red-ATP7B, green-Golgin 97, blue-DAPI, White scale bars on full tiled are 20 μM). Media were supplemented with 2 μM $CuCl_2$.
